# Society Meetings

**Published:** 1917-02

**Authors:** 


### SOCIETY MEETINGS

Brief reports and announcements of meetings of societies of Western New York. and those of general scope, are requested from Secretaries. Copy should be on hand the fifteenth of the month. Full transactions will be published at cost of composition.

**DOCTOR: Is your Society properly represented here? If not, please bring our standing announcement to the attention of the members.**

**The Medical Society of the County of Wyoming** met in Castile Jan. 11. Dr. A. B. Harding of Castile read a poem, "A Life with a Moral," Dr. N. G. Russell of Buffalo gave a paper on Nephritis, Dr. H. P. Cole one on Correction of Weakness and Deformity and their Correction, Dr. Harold E. Foster of Castile presented orthopaedic cases.

The annual dinner of the **Niagara County Medical Society** was held at the Kenmore Hotel, Lockport, Jan. 27, under the presidency of Dr. J. W. Corman of North Tonawanda.

**The Rochester Academy of Medicine,** Section 1, met Jan. 10, Robt. T. French Chairman, C. Clyde Sutter Secretary. David B. Jewett gave a paper on Focal Infection, Geo. W. Goler on Present Status of Serum Therapy.

During the meeting of the **American Congress on Internal Medicine** in N. Y. in December, the **American College of Physicians** was established and 65 fellows were admitted.

**The Rochester Academy of Medicine** held its annual meeting Jan. 10. Certain changes were made in the constitution and by-laws so as to have the same fiscal year for business purposes and the tenure of offices, ending in May instead of January. Officers were elected.

---

**The Buffalo Academy of Medicine:** Section of Pathology, Dec. 27, Use of X-Ray as a Diagnostic Factor, lantern slide demonstration, Leonard Reu.

Section of Surgery, Jan. 3, Modification of the Usual Club Foot Operation, with Report of Cases, Prescott Le Breton, discussion opened by Bernard Bartow.

Section of Medicine, Jan. 10, Prodromal or Medico-Legal Period of Paresis, Herman G. Matzinger; Treatment of Paresis by Swift-Ellis and Other Methods (lantern slides), John L. Eckel.

Stated meeting of Academy under charge of Section of Obstetrics and Gynaecology, Jan. 17, Appeal for Routine Wassermann Examination in Obstetric and Gynaecologic Hospital and Private Practice, Reuben Peterson, Ann Arbor. Collation served at close of meeting.

Section of Pathology, Jan. 24, Serum Therapy in Pneumonia, Augustus B. Wadsworth, M. D., Director of the State Dept. of Health Laboratory.

---

The annual meeting of the **Monroe County Homoeopathic Medical Society** was held on Tuesday evening, Jan. 9, in the auditorium of the Rochester Medical Association Building. The officers elected are W. Frank Fowler President, Lloyd H. Clark vice-president, Edmond R. Reynolds secretary and treasurer. The American Institute of Homoeopathy will meet in Rochester on June 17 to 23 inclusive as a guest of the Monroe County Homoepathic Society. A dinner was given in the association rooms in honor of Drs. Dearborn and Loizeaux of N. Y. Literary program—Some Recent Aspects of Cardio-Vascular-Renal Disease, Dr. David B. Jewett, discussion opened by Dr. Lucius L. Button; The Coming Institute Meeting, Dr. Frederick M. Dearborn, N. Y. City, discussion opened by Dr. John M. Lee; General Observations on the Workmen's Compensation Law, Dr. Harold H. Baker, discussion opened by Dr. Frank Bascom; A Discussion of Late Additions to Our Obstetric Literature, Dr. Leon S. Loizeaux, N. Y. City, discussion opened by Dr. William W. Winans.

